# A Biographical Sketch of the Life of Dr. T. B. Hamlin

**Published:** 1875-06

**Authors:** S. J. Cobb

**Affiliations:** Chairman of Committee on Memorials, Tennessee Dental Association.


### ARTICLE VI.

### *A Biographical Sketch of the Life of Dr. T. B. Hamlin, of Nashville, Tennessee.*

Dr. T. B. Hamlin was born June 24, 1810, at Red Hook, or Hamlin's Landing, on the Hudson River, Duchess Co., New York.

A few months after his birth, his father moved to Windom, west of the Catskill Mountains, where he died, leaving a family consisting of his wife and two sons in destitute circumstances. At the age of sixteen, his mother and brother died, leaving him quite alone. To be thus thrown upon his own resources so early in life, without money or education, was indeed a great misfortune to the object of this sketch, but he wisely and promptly bound himself for three years to a silversmith and watch repairer. During this time he conducted himself so well that his employer sent him to school for six months, and he also, in the latter part of his apprenticeship, received some little dental instruction from a physician who was practicing medicine and dentistry together in the little village in which he lived. At the age of nineteen, young Hamlin sought and obtained employment in one of the largest watch making establishments in the city of Albany, N. Y. His industry and proficiency soon led to his promotion to the responsible position of foreman of the watch making department. In his strenuous exertions to discharge the duties of this important position, he injured his health to such an extent that he was compelled to give up his lucrative and desirable situation and travel for the benefit of his health. After traveling and resting from business for some time, his health improved, and he was induced to return to Albany and resume his former position. His close confinement, however, soon told upon him, and he was forced by a renewal of his disease, to resign his situation finally From Albany he removed to Lee, Mass., where he opened a jewelry store. Shortly after, and at the age of twenty-two years, he married Miss Mary Phinney, a daughter of Auscher Phinney, a highly respectable citizen of that place. During Hamlin's stay in Lee, he was attacked with rheumatism, and this, in connection with his dyspepsia, caused him to look to the Sunny South for relief. In his travels he went to New Orleans, where he spent one winter, deriving great benefit to his health from the change of climate. While in New Orleans he invented an instrument or tool known as the centering or pivoting tool, now in use among all watch makers and repairers.

This little instrument has proved to be a very valuable invention, and if properly managed by the inventor, it might have resulted in great pecuniary profit to him.

From New Orleans, Hamlin moved to Wytheville, Va., where in 1835 he commenced the practice of dentistry, having studied the profession as opportunity offered, ever since he received his first instructions at Windom. Soon after he commenced the practice, he joined the American Society of Dental Surgeons, and ever after was a warm advocate of Dental Associations, and in 1840 he received an honorary diploma from the Baltimore Dental College, a graceful acknowledgment on the part of that institution of his worth and professional excellence.

After ten years practice in Wytheville and vicinity, he moved to Tuscumbia, Alabama, where he practiced his profession two years. In 1847 he removed to Nashville, Tenn., where he bought out Dr. Varcamp, paying him $6,000 for a three-story brick building on Cherry street, built expressly for a dental office. He immediately commenced the practice at Nashville, and for twelve or fifteen years did a large and lucrative business, profitable alike to himself and his patrons; to his patrons from the fact that he served them in such an honest, faithful and skillful manner, as to give them full value for every dollar they paid to him.

While Dr. Hamlin was not as extensively known in his profession as some others, no one stood higher among those who knew him well, and no one knew him well professionally or otherwise, who did not honor and respect him. Soon after he located in Nashville, he wrote and published a little book, called "Importance and Care of the Teeth," and shortly after he published a small work, called "Quackery Unwashed." These little unpretentious publications contained many valuable facts of much interest to the public as well as the profession, and we dare say much good was done by their general distribution.

During his practice in Nashville, he did a great deal in the way of interesting the people in the preservation of their teeth.

From the time he commenced the practice of dentistry, he labored with an eye single to the elevation of the dental profession, believing that to be the true way of elevating himself. The fact that he lived up to this high idea of duty, resulted in his great success, in his rising from obscurity to a very high and honorable position in his profession, and also in the accumulation of a very handsome property; the most of which was made in Nashville during the last fifteen years of his practice. In 1861, owing to ill-health, he closed his professional career, and after traveling four or five years through a portion of the United States and Canada, and regaining his strength to some extent, he returned to Nashville and purchased a farm some ten or twelve miles north of the city.

He immediately engaged with enthusiasm in the bee culture and nursery business. He soon became an accepted authority on the subject of bees, their habits, the best methods of treating them, etc., having been more or less interested in these little creatures many years before giving up his profession. He wrote a very interesting and instructive work on that subject, and received many premiums, medals and diplomas as testimonials of his accurate knowledge of the bee culture.

At the time of his death he owned one of the largest apiaries in the South.

Dr. Hamlin continued to zealously follow this new pursuit up the time of his death, which was on the 24th of May, 1874. He died in the 64th year of his age.

S. J. COBB,

*Chairman of Committee on Memorials,*

*Tennessee Dental Association.*

